# The Skin-Whitening and Antioxidant Effects of Protocatechuic Acid (PCA) Derivatives in Melanoma and Fibroblast Cell Lines

**DOI:** 10.3390/cimb45030138

**Published:** 2023-03-06

**Authors:** Jaehoon Cho, Hyeonbi Jung, Dong Young Kang, Nipin Sp, Wooshik Shin, Junhak Lee, Byung Gyu Park, Yoon A Kang, Kyoung-Jin Jang, Se Won Bae

**Affiliations:** 1 Green and Sustainable Materials R&D Department, Korea Institute of Industrial Technology (KITECH), Cheonan 31056, Republic of Korea; 2 Department of Chemistry and Cosmetics, Jeju National University, Jeju 63243, Republic of Korea; 3 Department of Pathology, School of Medicine, Institute of Biomedical Science and Technology, Konkuk University, Chungju 27478, Republic of Korea; 4 Department of Surgery, Division of Surgical Oncology, The Ohio State University Comprehensive Cancer Center, Columbus, OH 43210, USA; 5 R&D Center, ACTIVON Co., Ltd., Cheongju 28104, Republic of Korea

**Keywords:** B16 melanoma cells, HS68 fibroblast cells, protocatechuic acid derivatives, skin whitening, antioxidant

## Abstract

The skin is the most voluminous organ of the human body and is exposed to the outer environment. Such exposed skin suffers from the effects of various intrinsic and extrinsic aging factors. Skin aging is characterized by features such as wrinkling, loss of elasticity, and skin pigmentation. Skin pigmentation occurs in skin aging and is caused by hyper-melanogenesis and oxidative stress. Protocatechuic acid (PCA) is a natural secondary metabolite from a plant-based source widely used as a cosmetic ingredient. We chemically designed and synthesized PCA derivatives conjugated with alkyl esters to develop effective chemicals that have skin-whitening and antioxidant effects and enhance the pharmacological activities of PCA. We identified that melanin biosynthesis in B16 melanoma cells treated with alpha-melanocyte-stimulating hormone (α-MSH) is decreased by PCA derivatives. We also found that PCA derivatives effectively have antioxidant effects in HS68 fibroblast cells. In this study, we suggest that our PCA derivatives are potent ingredients for developing cosmetics with skin-whitening and antioxidant effects.

## 1. Introduction

The skin is the most voluminous layer of the human body, playing the critical role of being a protective barrier against environmental influences and maintaining homeostasis [1,2]. In addition, the skin plays a vital cosmetic role in the human body and protects against water loss of the body and environmental harm [3]. Many factors could contribute to aging skin, including extrinsic factors, such as sun exposure, air pollution, and smoking, and intrinsic factors, such as genetics. However, the aging of body organs is initiated at birth, and the skin is no exception. Skin aging is distinguished by pigmentation, sunspots, uneven skin color, loss of elasticity, wrinkling, and rough-textured appearance [4]. Therefore, skin care is quickly becoming important in terms of skin health and beauty.

Melanin is an essential factor in determining the skin, hair, and eye color of the body, playing a critical role in photoprotection through the absorption of solar ultraviolet radiation (UVR) [5,6]. In normal conditions, skin pigmentation by melanin synthesized in melanosomes plays the role of a photoprotective effect against UVR-induced DNA damage. In particular, α-melanocyte-stimulating hormone (α-MSH) induced by UVR exposure plays a leading role in protecting against DNA damage while mediating the induction of pigmentation in keratinocytes and melanocytes of skin cells [7,8,9]. However, hyperpigmentation by excessive production of melanin is a cause of dermatological problems, such as freckles, senile lentigo (age spots), melasma, melanoma, and post-inflammatory melanoderma [10,11,12]. The effect of skin whitening, known as skin lightening, is related to regulating the production of excessive melanin by performing melanogenesis-inhibitory activity and free radical scavenging capacity [13]. Therefore, various skin-whitening cosmetic agents are needed for dermatological treatment, skin beauty, and prevention.

Oxidative stress is a phenomenon caused by an imbalance between the production and accumulation of reactive oxygen species (ROS) in cells and tissues and the ability of a biological system to detoxify these reactive products [14,15,16]. In addition, oxidative stress plays a fundamental role in the pathogenesis of chronic diseases, such as neurodegenerative diseases, cancers, or infection by the human immunodeficiency virus (HIV), related to increased ROS production [17,18,19]. As already stated, skin aging progresses when one is born and is accelerated by oxidative stresses caused by intrinsic and extrinsic skin aging factors, such as hormonal changes, inflammation, lifestyle, smoking, and UVR [20]. Excessive oxidative stress in the skin is caused by inflammation, pigmentation, acne, blackhead, and melanoma [21]. Therefore, since oxidative stress is one of the significant causes of skin aging or skin disorders, it is crucial to properly neutralize it for dermal health, care, and beauty.

Plants are known as one of the sources used in the food, pharmaceutical, and cosmetic industries. Many products, such as supplements, nutricosmetics, and cosmetics are traditionally based on botanical ingredients [22]. Products of plant materials, including extracts, are used for the purpose of skin care and treatment of skin diseases, and they also contribute to the protection and restoration of skin barrier homeostasis [23]. Although the need for natural ingredients to make cosmetic products has increased, the effect of natural cosmetics, such as organic cosmetics, acts slower than conventional products. Some natural products could trigger allergic reactions. In addition, natural cosmetics may lack the exact substances that are needed for a particular type of skin. Thus, it is very important to use enough active ingredients with a specific efficacy in skin care. Recently, the main research trend has been the delivery of cosmetic substances for reforming cutaneous and subcutaneous layers of the skin in a healthy condition. In addition, there are many efforts to effectively improve the delivery of a specific natural compound by modifying the chemical structure [24,25]. The modified compounds generally undergo not only an efficacy evaluation but also a toxicity evaluation.

Protocatechuic acid (PCA), which is chemically known as 3,4-hydroxybenzoic acid, is one of the leading secondary metabolites of phenolic acid found in plant-based materials, such as vegetables and fruits. It is well known that PCA has antioxidative, anti-inflammatory, and osteoporotic activities that prevent aging-related diseases [26,27,28]. A study demonstrated that PCA suppresses LPS-induced inflammatory stress in BV2 microglia by regulating SIRT1/NF-κB pathway [29]. PCA showed an anti-inflammatory effect in LPS-stimulated BV2 microglia through the NF-κB and MAPK signaling pathway [30]. In addition, PCA has biological activities, such as anti-wrinkle and anti-skin-aging properties, through its antioxidative effects in vitro and in vivo [31]. Therefore, PCA is often used as a beauty ingredient in cosmetics with antioxidant and senescence-inhibiting activities [32]. Although it is becoming clear that PCA may be useful as a beauty intervention in the improvement of various human skin troubles, the potential of PCA derivatives in anti-melanogenesis and skin whitening as cosmetic ingredients has never been reported. One study revealed that PCA, modified for improving drug delivery, effectively has an anti-inflammatory effect for the therapy of osteoarthritis [33].

In this study, we chemically synthesized various PCA derivatives (e.g., PCA-C3, C4, C5, C6, C7, and C12) and conjugated them with alkyl esters. We purchased PCA-C0, C1, and C2 for comparative experiments. The PCA derivatives effectively inhibited the production of melanin induced by treatment with α-MSH in B16 melanoma cells. They also have been shown to have antioxidant activity similar to the effect of ascorbic acid, being a powerful antioxidant in HS68 fibroblast cells. We suggest that PCA derivatives are potent ingredients for skin-whitening and skin-care cosmetics.

## 2. Materials and Methods

### 2.1. Synthesis of PCA Derivatives

PCA-C0 (TCI C0055), C1 (TCI M1943), and C2 (D0571) were purchased from Tokyo Chemical Industry. Cellular tyrosinase activity was determined using a previously described method with modification. A series of alkyl esters of PCAs (e.g., PCA-C3, C4, C5, C6, and C7, and PCA-C12) were obtained by acid-catalyzed one-step esterification reaction utilizing dicyclohexylcarbodiimide (DCC) as an activating reagent, using a previously described method [34] and following a general procedure. Therefore, a solution of 3,4-dihydroxybenzoic acid (3.0 mmol) and alcohol (3.0 mmol) in THF (30 mL) cooled at 0°C was added to a solution of DCC (4.0 mmol). After the reaction mixture was stirred at room temperature, the solvent was partially evaporated under reduced pressure, and the residue was extracted with ethyl acetate three times. The combined organic layer was washed with brine, dried over sodium sulfate, and evaporated under reduced pressure. The resulting mixture was purified using fresh silica gel chromatography. The structures of the synthesized alkyl esters were characterized by ^1^H NMR. ^1^H NMR spectra were measured for acetone-d_6_ solutions at 25 °C using a JEOL JNM-ECX300 spectrometer, and the chemical shifts are reported in ppm.

PCA-C3: PCA-C3 was obtained in 34% yield as white power. 1H NMR (300 MHz, acetone-d_6_): δ 7.51 (d, J = 2.0 Hz, 1H), 7.47 (dd, J = 8.0, 2.0 Hz, 1H), 6.93 (d, J = 8.0 Hz, 1H), 4.32 (t, J = 7.2 Hz, 2H), 1.74 (sex, J = 7.2 Hz, 2H), 1.01 (t, J = 7.2 Hz, 3H).

PCA-C4: PCA-C4 was obtained in 40% yield as white power. 1H NMR (300 MHz, acetone- d_6_): δ 7.52 (d, J = 2.8 Hz, 1H), 7.45 (dd, J = 8.0, 2.8 Hz, 1H), 6.89 (d, J = 8.0 Hz, 1H), 4.23 (t, J = 7.2 Hz, 2H), 1.70 (quin, J = 7.2 Hz, 2H), 1.45 (sex, J = 7.2 Hz, 2H), 0.97 (t, J = 7.2 Hz, 3H).

PCA-C5: PCA-C5 was obtained in 46% yield as white power. 1H NMR (300 MHz, acetone- d_6_): δ 7.52 (d, J = 2.0 Hz, 1H), 7.46 (dd, J = 8.5, 2.0 Hz, 1H), 6.88 (d, J = 8.5 Hz, 1H), 4.24 (t, J = 7.0 Hz, 2H), 1.72 (quin, J = 7.0 Hz, 2H), 1.47 (m, 4H), 0.93 (t, J = 7.0 Hz, 3H).

PCA-C6: PCA-C6 was obtained in 48% yield as white power. 1H NMR (300 MHz, acetone- d_6_): δ 7.53 (d, J = 2.0 Hz, 1H), 7.45 (dd, J = 8.0, 2.0 Hz, 1H), 6.89 (d, J = 8.0 Hz, 1H), 4.23 (t, J = 6.8 Hz, 2H), 1.73 (quin, J = 6.8 Hz, 2H), 1.46 (m, 2H), 1.38 (m. 4H) 0.92 (t, J = 6.8 Hz, 3H).

PCA-C7: PCA-C7 was obtained at 37% yield as white power. 1H NMR (300 MHz, acetone- d_6_): δ 7.51 (d, J = 2.0 Hz, 1H), 7.48 (dd, J = 8.4, 2.0 Hz, 1H), 6.85 (d, J = 8.4 Hz, 1H), 4.24 (t, J = 6.8 Hz, 2H), 1.72 (quin, J = 6.8 Hz, 2H), 1.35 (m, 8H), 0.87 (t, J = 6.8 Hz, 3H).

PCA-C12: PCA-C12 was obtained in 58% yield as white power. 1H NMR (300 MHz, acetone- d_6_): δ 7.48 (d, J = 2.0 Hz, 1H), 7.43 (dd, J = 8.0, 2.0 Hz, 1H), 6.81 (d, J = 8.0 Hz, 1H), 4.23 (t, J = 6.8 Hz, 2H), 1.73 (quin, J = 6.8 Hz, 2H), 1.31 (m, 18H), 0.85 (t, J = 6.8 Hz, 3H).

### 2.2. Cell Culture and Cell Viability Assay

B16 F1 melanoma (No. 80007; Korean Cell Line Bank, Seoul, Korea) and HS68 fibroblast (CRL-1635; American Type Culture Collection, Manassas, VA, USA) cell lines were maintained in Dulbecco’s Modified Eagle Medium (Gibco; 22400-089) plus 10% FBS and 1% penicillin at 37 ℃ in 5% CO_2_. Cell viability was assayed by measuring the blue formazan that was metabolized from 3-(4,5-dimethylthiazol-2-yl)-2,5-diphenyl tetrazolium bromide (MTT) by mitochondrial dehydrogenase. The cells were resuspended in the medium one day before treatment at a density of 3 × 10^3^ cells per well in 96-well culture plates. The B16 and HS68 cells were incubated with or without various PCA derivative concentrations (e.g., C0, 1, 2, 3, 4, 5, 6, 7, and 12) for 24 h. MTT (5 mg/mL) was added to each well and incubated for 3 h at 37 °C. The formazan product was dissolved by adding DMSO, and absorbance was measured at 570 nm using an ultra-multifunctional microplate reader (TECAN, Durham, NC). All measurements were performed in triplicate and repeated at least three times.

### 2.3. Melanin Quantification Assay

B16 melanoma cells were seeded at 2 × 10^4^ cells/well and cultured in a 48-well plate for 24 h. The cell culture medium was changed, and the cells were treated with/without 200 nM of α-melanocyte-stimulating hormone (α-MSH) (SIGMA; M4135) and the indicated concentrations of protocatechuic acid (PCA) derivatives and further incubated at 37 °C for 72 h. After that, the supernatant of the culture medium was harvested, and the cells were gently washed three times with phosphate-buffered saline and then detached with trypsin/EDTA. The melanin contents of the supernatant and cells were lysed by incubation in 200 μL of 1 N NaOH at 80 °C for 2 h. The intracellular and extracellular melanin contents were transferred to a 96-well plate, and absorbance was measured at 405 nm. All measurements were performed in triplicate.

### 2.4. DPPH Antioxidant Assay

HS68 fibroblast cells were seeded at 3 × 10^3^ cells per well and cultured in 96-well culture plates for 24 h. Cells were treated with/without various indicated concentrations of PCA derivatives and further incubated at 37 °C for 48 h. The antioxidant effect of PCA derivatives was measured using a DPPH Antioxidant Assay Kit (Biovision; K2078) according to the manufacturer’s instructions. Absorbance was measured at 517 nm in endpoint mode at RT, protected from light. All measurements were conducted in triplicate.

### 2.5. Statistical Analyses

All experiments were conducted at least three times. The results are expressed as the mean ± standard error of the mean. Statistical analyses were performed using a one-way analysis of variance (*ANOVA*) with Tukey’s post hoc test using the SAS 9.3 software program (SAS Institute, Inc., Cary, NC, USA). A *p*-value < 0.05 (*) was considered to indicate a statistically significant difference (^#^indicates the significance level).

## 3. Results

### 3.1. Chemistry

**Figure 1:**
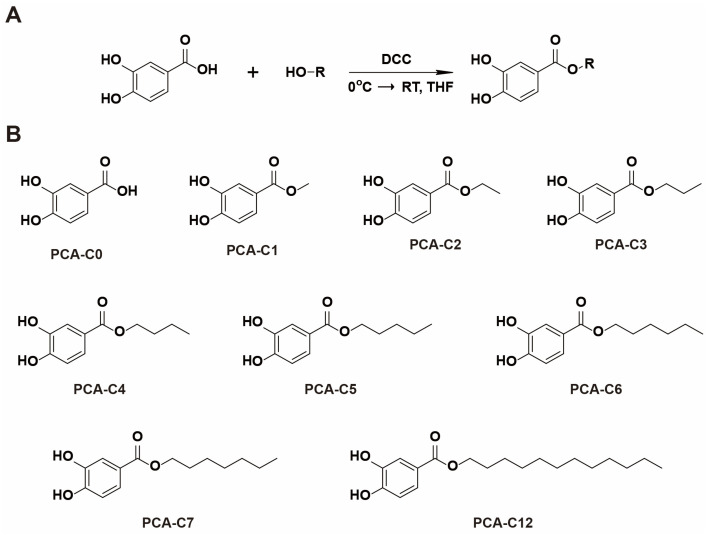
Molecular structures of PCA derivatives. (**A**) Synthetic procedure for alkyl ester derivatives of PCA. (**B**) A series of PCA derivatives: PCA-C0–C7 and C12.

As mentioned above, it is well known that PCA plays a role in various pharmacological activities, mainly attributable to its antioxidant and anti-inflammatory properties. It was demonstrated in one study that increased PCA hydrophobicity could improve anti-inflammatory activity and anti-mutagenicity [35]. Thus, we chemically synthesized PCA derivatives conjugated with different lengths of alkyl esters to change the hydrophobicity. For the synthesis of PCA-C3–C7 and C12, we used an acid-catalyzed one-step esterification reaction with dicyclohexylcarbodiimide (DCC) as an activating reagent (Figure 1A). Then, a series of PCA derivatives (PCA-C3–C7 and C12) was obtained for this study (Figure 1B and Figures S1–S6). 

### 3.2. Inhibitory Effect on α-MSH-Induced Melanogenesis Exhibited by PCA Derivatives in B16 Melanoma Cells

**Figure 2:**
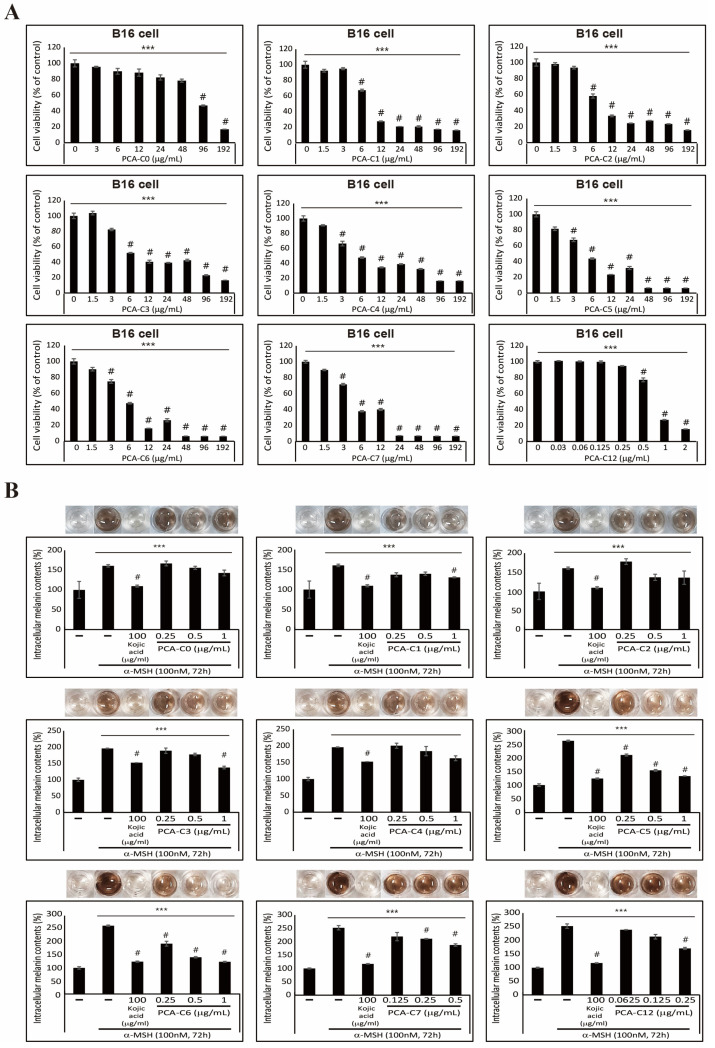
PCA derivatives inhibit α-MSH-induced melanin synthesis in B16 melanoma cells. (**A**) Cell viability results showed the inhibition of B16 cell proliferation after treatment with an increased PCA-C(X) concentration for 24 h. The data represent three independent tests. # *p* < 0.001 versus control. *** *p* < 0.001 (ANOVA test). (**B**) Intracellular melanin contents were determined using cultured media containing secreted melanin after α-MSH or/and PCA-C(X) treatment for 72 h. The results of extracellular melanin content can be found in Table S1. The pictures show the color of the culture medium, and the melanin content was reduced by PCA-C(X). The data are representative of three independent experiments. # *p* < 0.001 versus control. *** *p* < 0.001 (ANOVA test).

Before studying the anti-melanogenic effects of PCA derivatives, we hypothesized that the increased lengths of alkyl esters on PCA derivatives might increase cell apoptosis in B16 melanoma cells. So, we investigated their cytotoxicity by analyzing the cell proliferation of B16 melanoma cells in the presence of increasing concentrations of different PCA derivatives for 24 h (Figure 2A). These results imply that an increased hydrophobicity on PCA derivatives affects cell viability. Therefore, it was also indicated by the results of our cytotoxicity assay that the cells exhibit higher levels of cell death as the length of alkyl esters increases. In the PCA-C0–C6, cell viability was not affected by a concentration of less than 1 µg/mL, and PCA-C7 and C12 did not affect cell viability in concentrations lower than 1.5 µg/mL and 0.25 µg/mL. Next, we evaluated the inhibitory effects on α-MSH-induced melanin synthesis in B16 melanoma cells. We demonstrated that PCA derivatives show different inhibitory effects according to the length of the alkyl esters (Figure 2B). To evaluate the inhibitory effects of PCA derivatives, we performed a melanin quantification assay to investigate the extracellular or intracellular melanin content in the absence or presence of PCA derivatives in α-MSH-induced cells. It is well known that kojic acid inhibits melanin synthesis by inhibiting the tyrosinase enzyme [36]. So, we used kojic acid as a positive control in this study. It was shown in our results that PCA derivatives contribute to downregulating the extracellular and intracellular melanin contents compared to PCA-C0, which is not conjugated with alkyl esters in B16 melanoma cells (Figure 2B and Table S1). In particular, the treatment with 1 µg/mL of PCA-C5 and C6 significantly decreased the extracellular and intracellular melanin contents more than one-half-fold compared to only α-MSH-induced control. However, 1 µg/mL of PCA-C5 and C6 was the concentration without cytotoxicity to cells. It is indicated by these results that an increase in hydrophobicity of PCA derivatives due to conjugating alkyl esters increased the inhibitory effect of α-MSH-induced melanin synthesis. In addition, these data might mean that increased hydrophobicity improves the delivery of PCA-C5 and C6 into the cells. It has been reported in some studies that long-chain alkyl esters are frequently used to increase the biological activities of drugs [37,38]. 

### 3.3. Antioxidative Effect of PCA Derivatives on HS68 Fibroblast Cells

**Figure 3:**
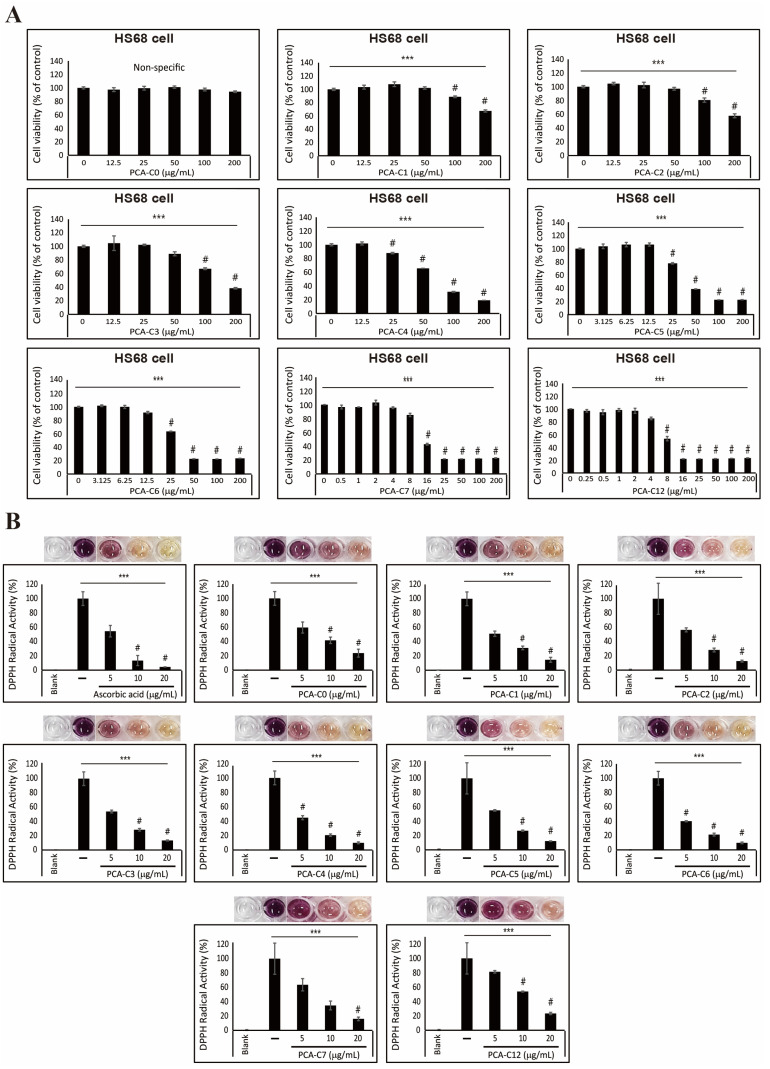
PCA derivatives have an antioxidant effect on HS68 fibroblast cells. (**A**) The inhibition of HS68 cell proliferation after treatment with an increased PCA-C(X) concentration for 24 h was shown by cell viability. The data represent three independent tests. # *p* < 0.001 versus control. *** *p* < 0.001 (ANOVA test). (**B**) The antioxidant effect of the PCA derivatives was confirmed by measuring DPPH radical activity. The pictures show that the color of the culture medium was reduced by PCA-C(X). The data are representative of three independent experiments. # *p* < 0.001 versus control. *** *p* < 0.001 (ANOVA test).

**Figure 4:**
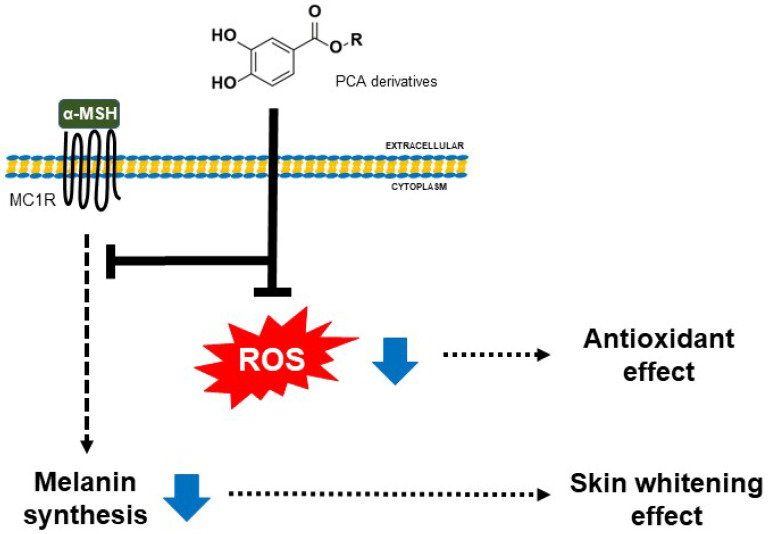
A simple scheme of anti-melanogenesis and antioxidant effects exerted by PCA derivatives. The anti-whitening effect of PCA derivatives is mediated by inhibiting the melanin synthesis induced via α-melanocyte-stimulating hormone (α-MSH) in B16 melanoma cells. In addition, PCA derivatives have a more effective antioxidant effect than PCA in HS68 fibroblast cells. We suggest that PCA derivatives might have multiple effects for other needs.

We first investigated the cell proliferation of HS68 fibroblast cells in an increasing concentration of PCA derivatives to assess the cytotoxicity and evaluate the antioxidative ability of PCA derivatives (Figure 3A). Although PCA-C0, which is not conjugated with alkyl esters, did not influence cell viability, despite the treatment with a high concentration, treatment with PCA derivatives decreased cell viability with an increase in their concentrations. Notably, PCA-C7 and C12 had high cytotoxicity in HS68 fibroblast cells, which means increased hydrophobicity due to conjugating alkyl ester chains enhances cell death. We treated HS68 fibroblast cells with 5, 10, or 20 µg/mL of PCA derivatives for 24 h to determine antioxidant effects. We evaluated the free radical scavenging ability of PCA derivatives using a DPPH radical scavenging assay. Although PCA-C0 resulted in a decrease in free radical activity, PCA derivatives, especially PCA-C5 and C6, effectively decreased free radicals, showing an antioxidant effect (Figure 3B). In particular, PCA-C6 had the most effective antioxidant activity compared to ascorbic acid as a positive control. These results indicate that the conjugation of alkyl esters to PCA increased the hydrophobicity of PCA and that its characteristic enhanced an increase in the antioxidant activity in HS68 fibroblast cells. We suggest that PCA derivatives could contribute to anti-melanogenesis and antioxidant effects (Figure 4).

## 4. Discussion

PCA is a natural phenolic compound well known for its antioxidative, anti-inflammatory, and anti-osteoporotic activities [26,27,28]. For example, PCA could protect against cell damage, apoptosis, and renal ischemia–reperfusion injury by reducing oxidative stress and tissue damage in rat cardiac muscle [39,40]. In addition, it was described in some studies that excessive inflammation, which can induce diseases such as arthritis, type 2 diabetes, and different types of cancer, was reduced by PCA [41,42]. It was clarified in one study that PCA compounds have biological activities, such as antioxidant activity, an anti-wrinkle effect, and collagen synthesis, as cosmetic ingredients [31]. However, the use of PCA as an anti-skin-aging ingredient is not suitable due to the high dose required. In this study, we chemically synthesized PCA derivatives containing alkyl esters of different lengths to lower their effective concentrations. We showed that our PCA derivatives have a cosmeceutical effect via skin-whitening and antioxidant activities at low concentrations. 

Melanogenesis is the process of the production of melanin pigment, which is produced in melanosomes by melanocytes. The process contains a series of enzymatic and chemical reactions in melanosomes, producing two types of melanin: eumelanin and pheomelanin [5,26,36,43]. α-MSH is a primary melanocyte-stimulating hormone that stimulates melanogenesis triggered by UVR exposure. α-MSH binds to the melanocortin 1 receptor (MC1R), which induces the activation of MCIR downstream, the cAMP/CREB signaling pathway for melanin production in melanocytes [26,44,45]. Various skin-whitening compounds extracted from natural products exert their effects by regulating the production of melanin through many mechanisms, including inhibiting the core signaling pathway and suppressing melanogenic gene expression [36]. One study demonstrated that [6]-shogaol, which is the major shogaol in the ginger rhizome, inhibits α-MSH-induced melanogenesis through the acceleration of ERK- and PI3K/AKT-mediated MITF degradation [45]. In addition, a PCA extracted from pears suppressed the conversion of ATP to cAMP downstream of MC1R, which resulted in the inhibition of melanogenesis by inhibiting melanogenic gene expression and melanin synthesis in B16F10 cells. We chemically synthesized PCA derivatives containing alkyl esters of various lengths to improve PCA delivery. These PCA derivatives, especially PCA-C5 and C6, effectively inhibited melanin synthesis at low concentrations as the lengths of alkyl esters increased (Figure 1 and Figure 2). However, PCA-C7 and PCA-C12 had cytotoxicity, although melanin synthesis had an inhibitory effect.

ROS, often referred to as free radicals, such as superoxide anion radical (O_2_^−•^) and hydroxyl radical (^•^OH), are harmful to the skin [46]. ROS accumulated in the body induce oxidative stress in the skin. Eventually, ROS cause various skin disorders, such as acne, blackhead, and melanoma [21]. The generation of oxidative stress in the skin is usually caused by UV/visible light/infrared radiation, pollution, skin microbiota, and stress. It was revealed in some studies that single compounds extracted from natural products had antioxidant effects on the skin. A natural compound extracted from *Centella asiatica* (L.) had antioxidant effects by elevating the transcription of antioxidant enzymes, such as CAT, GPx1, SOD1, and SOD2, and inhibiting the expression of an MMP9 transcript in human foreskin fibroblasts [47]. In addition, it was demonstrated in another study that natural compounds extracted from *Artemisia iwayomogi* (Dowijigi) decrease UV-mediated oxidative stress [48]. As mentioned above, it is well known that PCA has an antioxidant effect on the body. One group reported that PCA plays a role in oxidative scavenging stress from UVA irradiation damage in human dermal fibroblasts [31]. In our results, we indicated that PCA derivatives, especially PCA-C6, conjugated with alkyl esters of various lengths decrease oxidative stress more effectively than PCA-C0 in the HS68 fibroblast cells (Figure 3). It was indicated in our results that PCA derivatives chemically synthesized with alkyl esters have the potential to scavenge free radicals as the alkyl esters are longer. Many natural-derived substances have been used for the purpose of skin beauty or treatment [49]. Although synthetic or semi-synthetic substances have a lower safety than natural sources with non-toxicity and fewer side effects [50,51], they are used for cosmetic formulations of various purposes. We know that PCA derivatives presented in this study need to undergo the additional investigations for efficacy and safety through in vivo testing. However, we carefully suggest that alkyl ester-conjugated PCA derivatives can be applied as anti-skin-aging ingredients of cosmeceuticals at low concentrations.

## 5. Conclusions

Protocatechuic acid (PCA), a type of natural phenolic acid, has been known to have not only an antioxidant effect but also various biological properties, such as antibacterial, anticancer, antiviral, and anti-inflammatory effects in vitro and in vivo. In this study, we investigated the anti-melanogenesis or skin-whitening and antioxidant effects of synthesized PCA derivatives. We chemically synthesized PCA derivatives conjugated with alkyl esters of different lengths. We performed a cell cytotoxicity assay for deciding effective concentrations. Then, we demonstrated that PCA derivatives, especially PCA-C5 and C6, more effectively suppress the melanin biosynthesis in the B16 melanoma cells and inhibit the free radical activity in the HS68 fibroblast cells at low concentrations than only PCA. It can be concluded that our PCA derivatives are potentially effective anti-skin-aging ingredients for cosmeceuticals. Our results suggest that PCA derivatives conjugated with alkyl esters will be used to develop cosmetics with various effects.

## Data Availability

Not applicable.

## Supplementary Materials

The following supporting information can be downloaded at: https://www.mdpi.com/article/10.3390/cimb45030138/s1, Figure S1: ^1^H NMR spectra for PCA-C3; Figure S2: ^1^H NMR spectra for PCA-C4; Figure S3: ^1^H NMR spectra for PCA-C5; Figure S4: ^1^H NMR spectra for PCA-C6; Figure S5: ^1^H NMR spectra for PCA-C7; Figure S6: ^1^H NMR spectra for PCA-C12; Table S1: PCA derivatives reduce α-MSH-mediated melanin synthesis in B16 melanoma cells.
