# Supplementary material for: The Skin-Whitening and Antioxidant Effects of Protocatechuic Acid (PCA) Derivatives in Melanoma and Fibroblast Cell Lines
Source: Curr Issues Mol Biol. 2023 Mar 6;45(3):2157–69. doi: 10.3390/cimb45030138 (PMC10047566; doi:10.3390/cimb45030138)
Supplement: Supplementary file 1 [file cimb-45-00138-s001.zip › cimb-2169903-supplementary.pdf]

Supplementary file

The Skin-Whitening and Antioxidant Effects of Protocatechuic Acid (PCA) Derivatives in Melanoma and Fibroblast Cell Lines

Jaehoon Cho [1,†], Hyeonbi Jung [2,†], Dong Young Kang [3,‡], Nipin Sp [4], Wooshik Shin [1], Junhak Lee [5], Byung Gyu Park [5], Yoon A Kang [2], Kyoung-Jin Jang [3,‡,§,*] and Se Won Bae [2,*]

[1] Green and Sustainable Materials R&D Department, Korea Institute of Industrial Technology (KITECH),
Cheonan 31056, Republic of Korea; cjh0107@kitech.re.kr (J.C.); sws@kitech.re.kr (W.S.)

[2] Department of Chemistry and Cosmetics, Jeju National University, Jeju 63243, Republic of Korea; gusql8309@jejunu.ac.kr (H.J.); kya9887@jejunu.ac.kr (Y.A.K.)

[3] Department of Pathology, School of Medicine, Institute of Biomedical Science and Technology,
Konkuk University, Chungju 27478, Republic of Korea; kdy6459@kku.ac.kr

[4] Department of Surgery, Division of Surgical Oncology, The Ohio State University Comprehensive Cancer Center, Columbus, OH 43210, USA; sree04@osumc.edu

[5] R&D Center, ACTIVON Co., Ltd., Cheongju 28104, Republic of Korea; jhlee1708@activon.kr (J.L.); bgpark1503@activon.kr (B.G.P.)

* Correspondence: jangkj@konkuk.ac.kr (K.-J.J.); swbae@jejunu.ac.kr (S.W.B.)

† These authors contributed equally to this work.

‡ Current address: Department of Immunology, School of Medicine, Konkuk University, 268
Chungwon-daero, Chungju 27478, Republic of Korea.

§ Current address: Institute of Biomedical Science and Technology, Konkuk University, 120 Neungdong-ro, Gwangjin-gu, Seoul 05029, Republic of Korea.

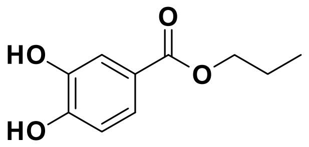

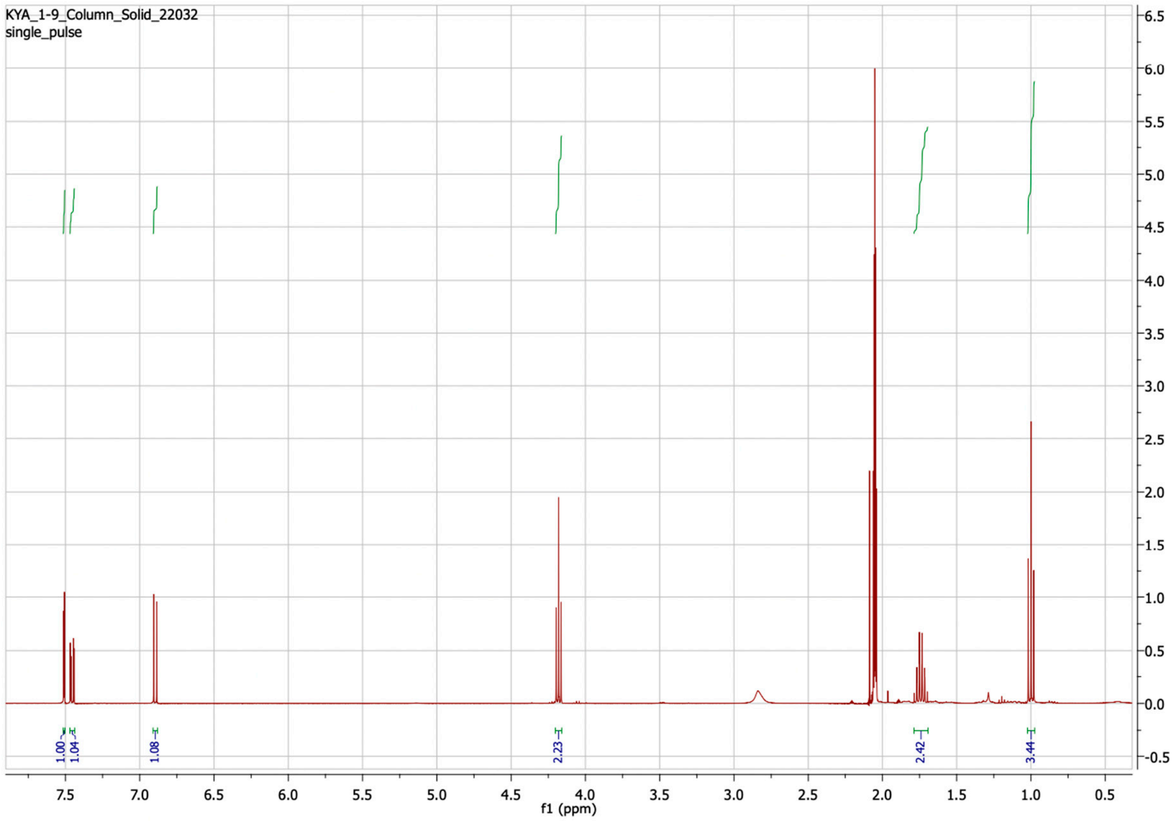

Figure S1. ^{1}H NMR spectra for PCA-C3

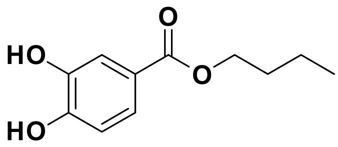

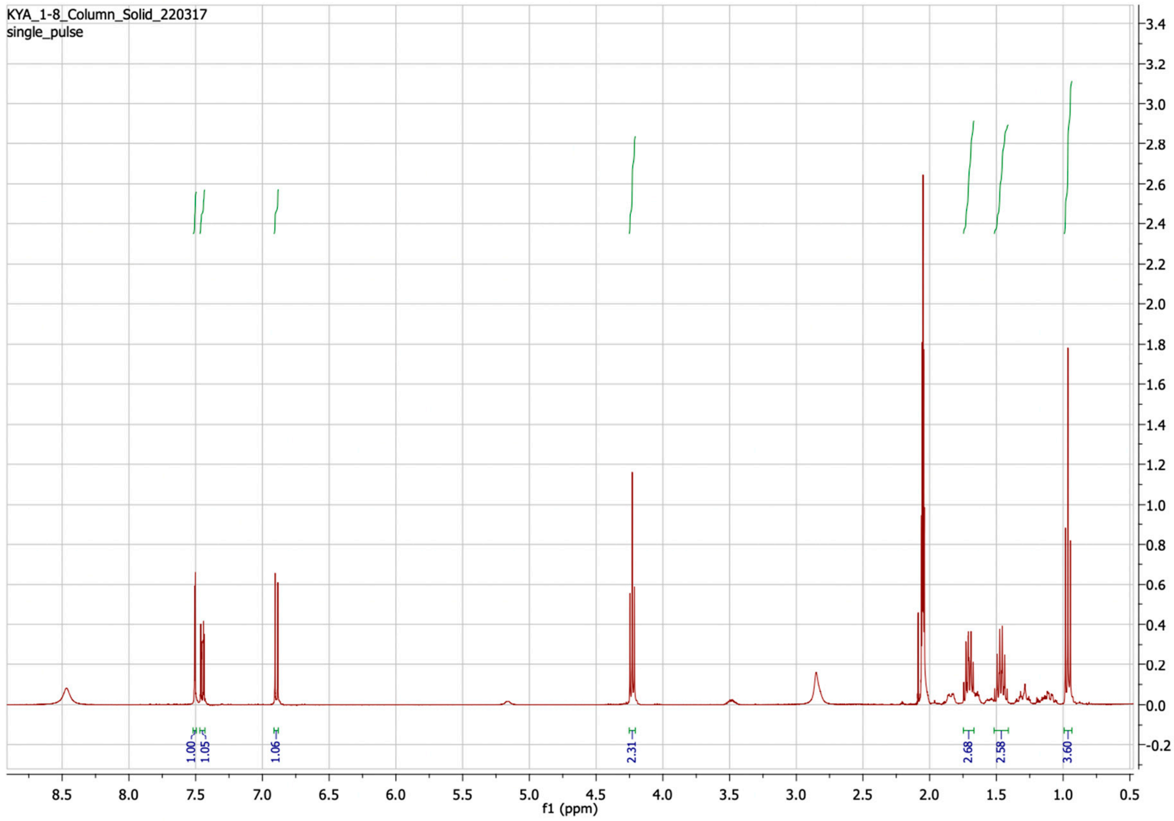

Figure S2. ^{1}H NMR spectra for PCA-C4

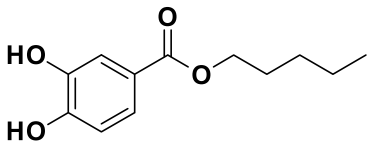

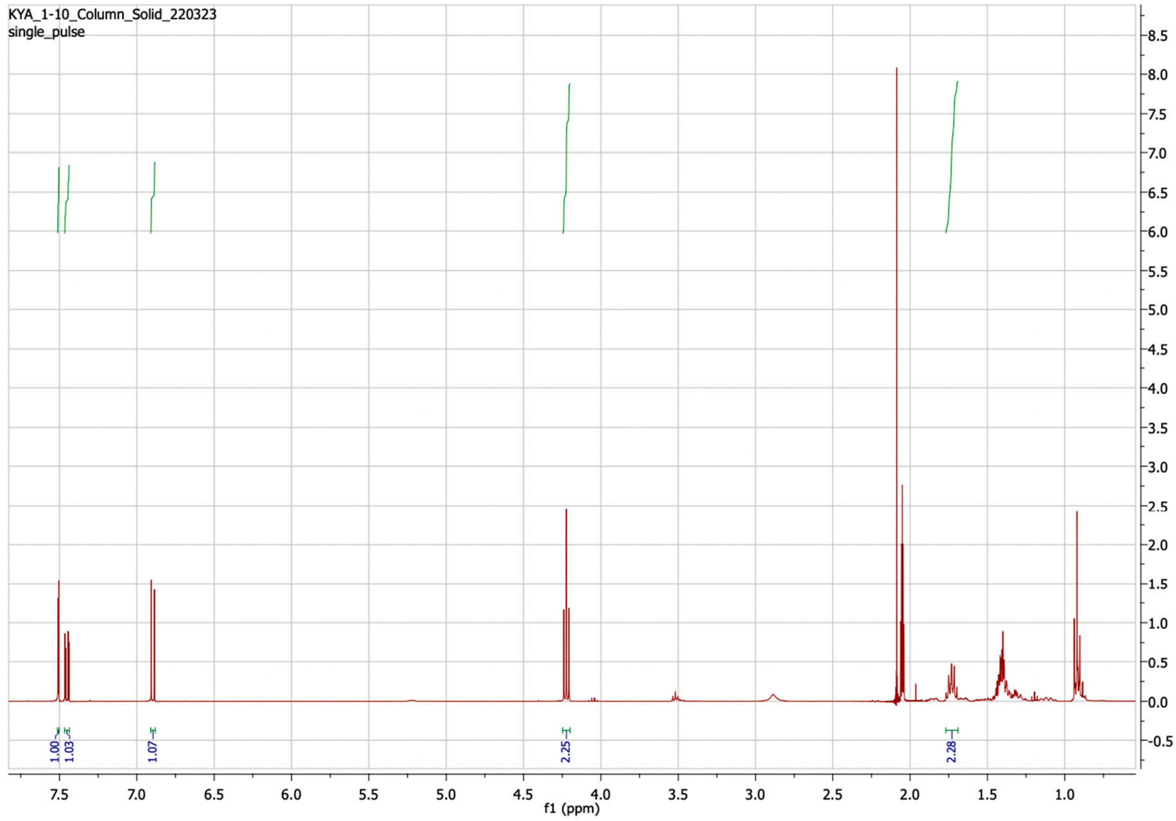

Figure S3. ¹H NMR spectra for PCA-C5

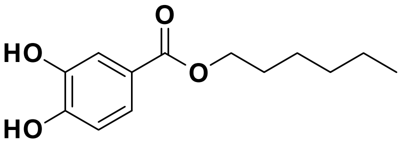

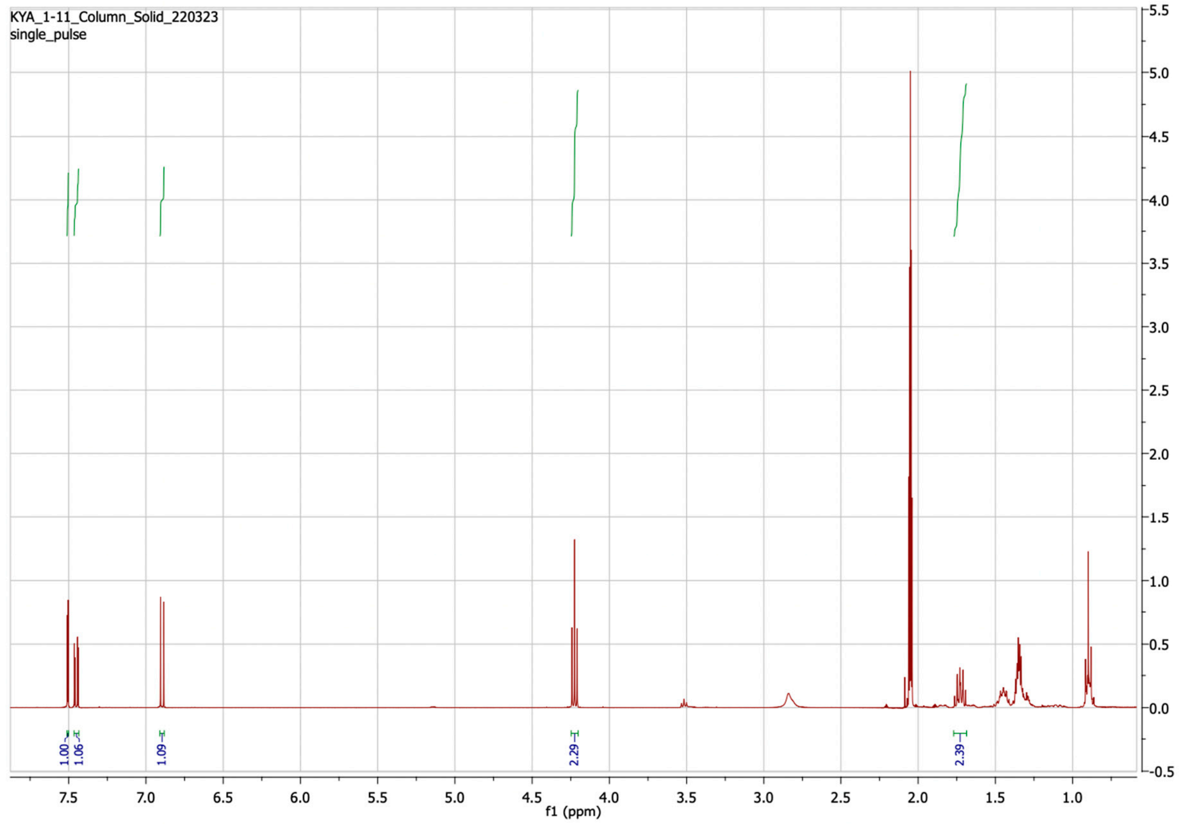

Figure S4. **¹H NMR spectra for PCA-C6**

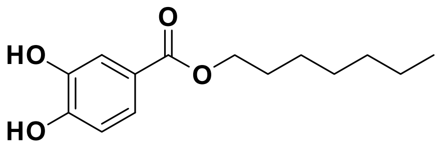

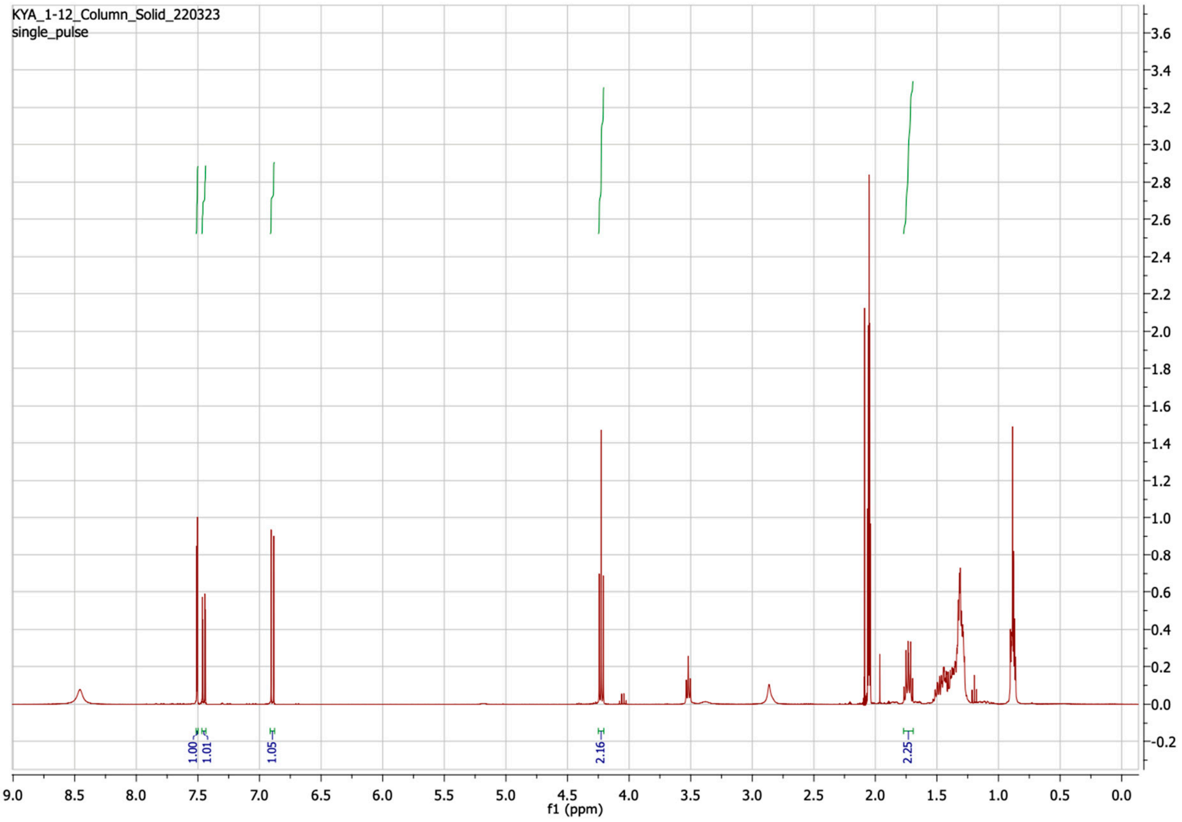

Figure S5. ^{1}**H NMR spectra for PCA-C7**

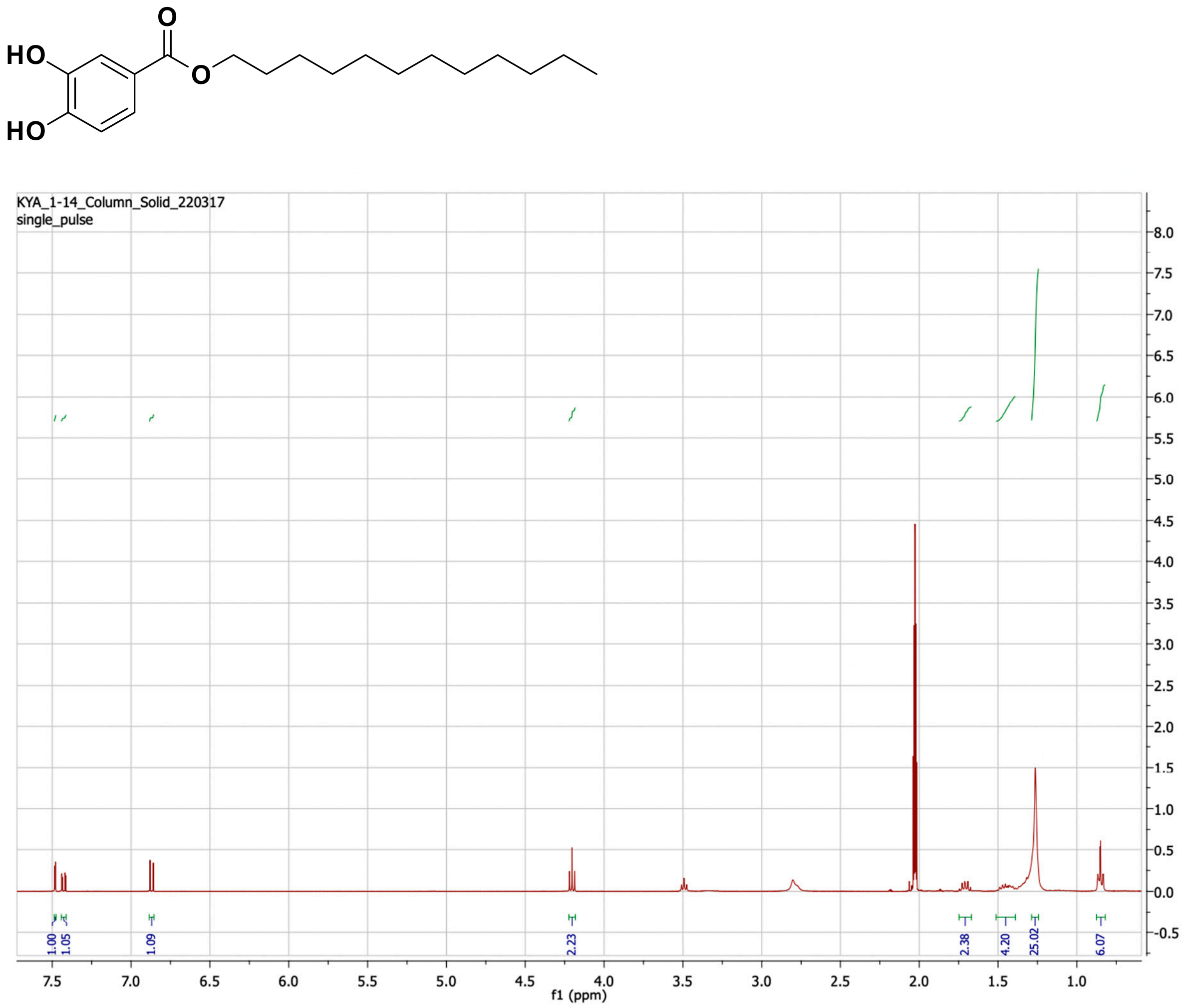

Figure S6. ^{1}H NMR spectra for PCA-C12

Table S1. PCA derivatives reduce α-MSH-mediated melanin synthesis in B16 melanoma cells.

			Extracellular Melanin Content		Intracellular Melanin Content	
Compound		Conc. (μg/ml)	Inhibition rate (%)	P-value	Inhibition rate (%)	P-value
α-MSH (100nM, 72h)	Kojic acid	100	55 ± 2.56	0.0031	84 ± 4.37	0.4867
	PCA-C0	0.25	≤ 5	0.0003	≤ 5	0.0066
		0.5	≤ 5	0.0027	8 ± 6.55	0.0114
		1	≤ 5	0.0002	30 ± 11.98	0.0308
	PCA-C1	0.25	≤ 5	0.0006	39 ± 8.06	0.0429
		0.5	31 ± 12.11	0.0021	35 ± 6.99	0.0347
		1	40 ± 11.16	0.0031	50 ± 1.92	0.0689
	PCA-C2	0.25	19 ± 1.23	0.0003	≤ 5	0.0040
		0.5	16 ± 6.77	0.0004	39 ± 13.14	0.0492
		1	24 ± 2.56	0.0245	41 ± 28.75	0.0734
	PCA-C3	0.25	≤ 5	0.0001	7 ± 8.68	0.0001
		0.5	27 ± 5.45	0.0000	19 ± 3.92	0.0000
		1	46 ± 5.03	0.0001	61 ≤ 4.33	0.0006
	PCA-C4	0.25	20 ± 1.15	0.0000	≤ 5	0.0000
		0.5	24 ± 11.13	0.0003	12 ± 14.48	0.0006
		1	50 ± 2.40	0.0000	35 ± 7.78	0.0003
	PCA-C5	0.25	32 ± 2.24	0.0000	17 ± 19.60	0.2384
		0.5	67 ± 1.88	0.0000	51 ± 9.15	0.0016
		1	80 ± 0.43	0.0000	72 ± 7.76	0.0003
	PCA-C6	0.25	42 ± 5.70	0.0002	≤ 5	0.3885
		0.5	75 ± 1.49	0.0000	64 ± 7.42	0.0005
		1	86 ± 1.14	0.0000	≥ 95	0.0001
	PCA-C7	0.125	22 ± 10.03	0.0298	≤ 5	0.0044
		0.25	27 ± 0.76	0.0010	≤ 5	0.0021
		0.5	42 ± 2.86	0.0003	≤ 5	0.0007
	PCA-C12	0.0625	9 ± 0.44	0.0456	66 ± 48.44	0.0950
		0.125	25 ± 5.31	0.0042	≤ 5	0.0238
		0.25	53 ± 2.43	0.0001	≤ 5	0.1421
